# Genome sequence of the free-living aerobic spirochete *Turneriella parva* type strain (H^T^), and emendation of the species *Turneriella parva*

**DOI:** 10.4056/sigs.3617113

**Published:** 2013-05-25

**Authors:** Erko Stackebrandt, Olga Chertkov, Alla Lapidus, Matt Nolan, Susan Lucas, Nancy Hammon, Shweta Deshpande, Jan-Fang Cheng, Roxanne Tapia, Lynne A. Goodwin, Sam Pitluck, Konstantinos Liolios, Ioanna Pagani, Natalia Ivanova, Konstantinos Mavromatis, Natalia Mikhailova, Marcel Huntemann, Amrita Pati, Amy Chen, Krishna Palaniappan, Miriam Land, Chongle Pan, Manfred Rohde, Sabine Gronow, Markus Göker, John C. Detter, James Bristow, Jonathan A. Eisen, Victor Markowitz, Philip Hugenholtz, Tanja Woyke, Nikos C. Kyrpides, Hans-Peter Klenk

**Affiliations:** 1 Leibniz-Institute DSMZ - German Collection of Microorganisms and Cell Cultures, Braunschweig, Germany; 2 Los Alamos National Laboratory, Bioscience Division, Los Alamos, New Mexico, USA; 3 DOE Joint Genome Institute, Walnut Creek, California, USA; 4 Biological Data Management and Technology Center, Lawrence Berkeley National Laboratory, Berkeley, California, USA; 5 Oak Ridge National Laboratory, Oak Ridge, Tennessee, USA; 6 HZI – Helmholtz Centre for Infection Research, Braunschweig, Germany; 7 University of California Davis Genome Center, Davis, California, USA; 8 Australian Centre for Ecogenomics, School of Chemistry and Molecular Biosciences, The University of Queensland, Brisbane, Australia

**Keywords:** Gram-negative, motile, axial filaments, helical, flexible, non-sporulating, aerobic, mesophile, *Leptospiraceae*, GEBA

## Abstract

*Turneriella parva* Levett *et al*. 2005 is the only species of the genus *Turneriella* which was established as a result of the reclassification of *Leptospira parva* Hovind-Hougen *et al*. 1982. Together with *Leptonema* and *Leptospira*, *Turneriella* constitutes the family *Leptospiraceae*, within the order *Spirochaetales*. Here we describe the features of this free-living aerobic spirochete together with the complete genome sequence and annotation. This is the first complete genome sequence of a member of the genus *Turneriella* and the 13^th^ member of the family *Leptospiraceae* for which a complete or draft genome sequence is now available. The 4,409,302 bp long genome with its 4,169 protein-coding and 45 RNA genes is part of the *** G****enomic* ***E****ncyclopedia of* ***Bacteria**** and* ***Archaea***** project.

## Introduction

Strain H^T^ (= DSM 21527 = NCTC 11395 = ATCC BAA-1111) is the type strain of *Turneriella parva* [1]. The strain was isolated from contaminated *Leptospira* culture medium [2] and was originally thought to be affiliated with *Leptospira* [2] because of morphological similarities to other members of the genus. Strain H^T^ was designated as a separate species because of certain morphological and molecular differences: cells were shorter and were more tightly wound, the surface layer formed blebs instead of cross-striated tubules when detached for negative staining preparation and the base composition of DNA differed from that of other *Leptospira* species [2]. DNA-DNA hybridization [3] and enzyme activity [4] studies revealed sufficient differences between other *Leptospira* species and *L. parva* that the ‘Subcommittee on the Taxonomy of *Leptospira*’ [5] decided to exclude *L. parva* from the genus *Leptospira* and assign it as the type strain of a new genus: ‘*Turneria’* as ‘*Turneria parva*’. The genus was named in honor of Leslie Turner, an English microbiologist who made definitive contributions to the knowledge of leptospirosis [1]. However, as the generic name is also in use in botany and zoology, this name was rendered illegitimate and invalidate, but was used in the literature [6,7]. The first 16S rRNA gene-based study (Genbank accession number Z21636), performed on *Leptospira parva incertae sedis,* confirmed the isolated position of *L. parva* among *Leptonema* and *Leptospira* species [8], a finding later supported by Morey *et al*. [9]. The reclassification of *L. parva* as *Turneriella parva* com. nov. was published by Levett *et al.* [1], reconfirming the separate position of the type strain [10] and an additional strain (S-308-81, ATCC BAA-1112) from the uterus of a sow from all other leptospiras on the basis of DNA-DNA hybridization and 16S rRNA gene sequence analysis (Genbank accession number AY293856). The strain was selected for genome sequencing because of its deep branching point within the *Leptospiraceae* lineage.

Here we present a summary classification and a set of features for *T. parva* H^T^ together with the description of the complete genomic sequencing and annotation.

## Classification and features

### 16S rRNA gene sequence analysis

A representative genomic 16S rDNA sequence of *T. parva* H^T^ was compared using NCBI BLAST [11,12] under default settings (e.g., considering only the high-scoring segment pairs (HSPs) from the best 250 hits) with the most recent release of the Greengenes database [13] and the relative frequencies of taxa and keywords (reduced to their stem [14]) were determined, weighted by BLAST scores. The most frequently occurring genera were *Geobacter* (48.7%), *Leptospira* (19.2%), *Pelobacter* (13.4%), *Spirochaeta* (8.1%) and *Turneriella* (6.4%) (56 hits in total). Regarding the single hit to sequences from members of the species, the average identity within HSPs was 95.8%, whereas the average coverage by HSPs was 89.8%. Among all other species, the one yielding the highest score was *Leptonema illini* (AY714984), which corresponded to an identity of 85.7% and an HSP coverage of 62.6%. (Note that the Greengenes database uses the INSDC (= EMBL/NCBI/DDBJ) annotation, which is not an authoritative source for nomenclature or classification.) The highest-scoring environmental sequence was DQ017943 (Greengenes short name 'Cntrl Erpn Rnnng Wtrs Exmnd TGGE and uplnd strm cln S-BQ2 83'), which showed an identity of 95.6% and an HSP coverage of 97.8%. The most frequently occurring keywords within the labels of all environmental samples which yielded hits were 'microbi' (5.5%), 'sediment' (2.6%), 'soil' (2.5%), 'industri' (2.1%) and 'anaerob' (1.9%) (194 hits in total). Environmental samples which yielded hits of a higher score than the highest scoring species were not found.

**Figure 1:**
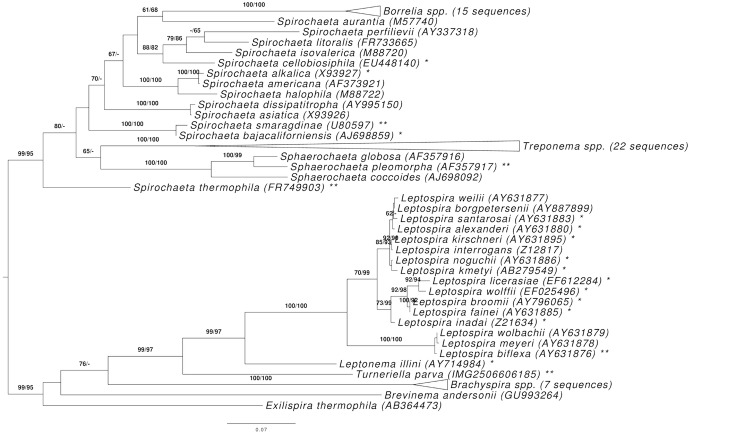
Phylogenetic tree highlighting the position of *T. parva* relative to the type strains of the other species within the phylum *'Spirochaetes'*. The tree was inferred from 1,318 aligned characters [15,16] of the 16S rRNA gene sequence under the maximum likelihood (ML) criterion [17]. Rooting was done initially using the midpoint method [18] and then checked for its agreement with the current classification (Table 1). The branches are scaled in terms of the expected number of substitutions per site. Numbers adjacent to the branches are support values from 500 ML bootstrap replicates [19] (left) and from 1,000 maximum-parsimony bootstrap replicates [20] (right) if larger than 60%. Lineages with type strain genome sequencing projects registered in GOLD [21] are labeled with one asterisk, those also listed as 'Complete and Published' with two asterisks [22-28]; for *Sphaerochaeta pleomorpha* see CP003155. The collapsed *Treponema* subtree contains three species formerly assigned to *Spirochaeta* that have recently been included in the genus *Treponema*, even though those names are not yet validly published [27].

Figure 1 shows the phylogenetic neighborhood of *T. parva* H^T^ in a 16S rRNA based tree. The sequences of the two identical 16S rRNA gene copies in the genome do not differ from the previously published 16S rRNA sequence (AY293856).

**Table 1:** Classification and general features of *T. parva* H^T^ according to the MIGS recommendations [29].

**MIGS ID**	**Property**	**Term**	**Evidence code**
		Domain *Bacteria*	TAS [30]
		Phylum *Spirochaetes*	TAS [31]
		Class *Spirochaetes*	TAS [32,33]
	Current classification	Order *Spirochaetales*	TAS [34,35]
		Family *Leptospiraceae*	TAS [1,35,36]
		Genus *Turneriella*	TAS [1]
	Species	*Turneriella parva*	TAS [1]
MIGS-7	Subspecific genetic lineage (strain)	*Turneriella parva* H^T^	TAS [1]
MIGS-12		Levett *at al*. 2005	TAS [1]
	Gram stain	negative	TAS [1]
	Cell shape	spiral-shaped	TAS [1]
	Motility	motile	TAS [1]
	Sporulation	non-sporulating	
	Temperature range	mesophile	TAS [1]
	Optimum temperature	grows between 11 and 37 °C	TAS [1]
	Salinity	not reported	
MIGS-22	Relationship to oxygen	aerobe	TAS [1]
	Carbon source	long-chain fatty acids and long-chain alcohols	TAS [4]
	Energy metabolism	chemoheterotrophic	TAS [4]
MIGS-6	Habitat	not reported	
MIGS-6.2	pH	not reported	
MIGS-15	Biotic relationship	free living	TAS [1]
MIGS-14	Known pathogenicity	not reported	
MIGS-16	Specific host	not reported	
MIGS-18	Health status of host	unknown	
	Biosafety level	1	TAS [37]
MIGS-19	Trophic level	unknown	
MIGS-23.1	Isolation	contaminated culture medium	TAS [1]
MIGS-4	Geographic location	Regina, Saskatchewan, Canada	TAS [1]
MIGS-5	Time of sample collection	1981	TAS [1]
MIGS-4.1	Latitude	50.45	TAS [1]
MIGS-4.2	Longitude	-104.61	TAS [11]
MIGS-4.3	Depth		
MIGS-4.4	Altitude		

Evidence codes - TAS: Traceable Author Statement (i.e., a direct report exists in the literature); NAS: Non-traceable Author Statement (i.e., not directly observed for the living, isolated sample, but based on a generally accepted property for the species, or anecdotal evidence). Evidence codes are from the Gene Ontology project [38].

### Morphology and physiology

**Figure 2:**
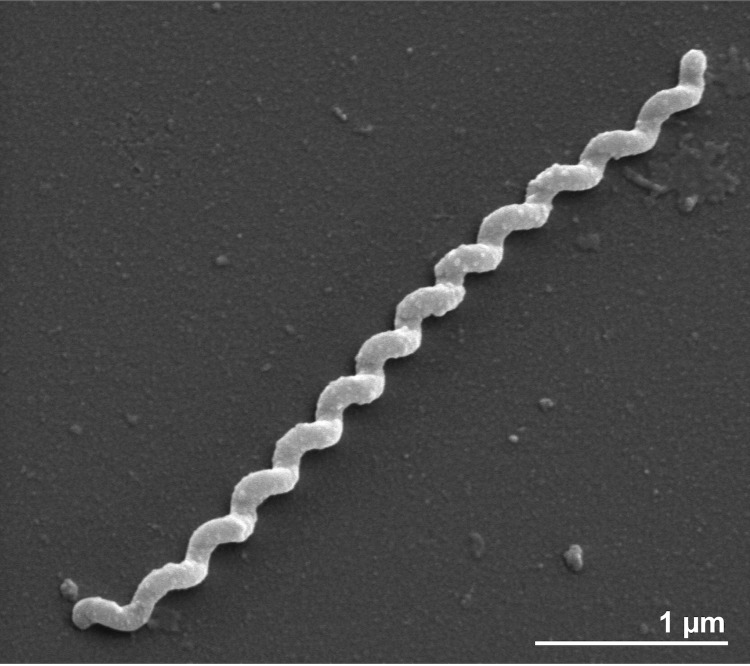
Scanning electron micrograph of *T. parva* H^T^

Cells of strain H^T^ are Gram-negative, flexible and helical with 0.3 µm in diameter and 3.5-7.5 µm in length and a wavelength of 0.3-0.5 µm (Figure 2). Motility is achieved by means of two axial filaments, similar to those of other leptospiras. The surface of the cells show several blebs with no apparent substructure when prepared for negative staining while under the same conditions, cross-striated tubules are visible in other leptospiras [1,2]. The strain is obligately aerobic and oxidase positive. Slow and limited growth occurs in polysorbate albumin medium [39] at 11, 30 and 37 °C. Growth is inhibited by 8-azaguanine (200 µg ml^-1^) and 2,6 diaminopurine (µg ml^-1^). Lipase is produced, long-chain fatty acids and long-chain fatty alcohols are utilized as carbon and energy sources. L-lysine arylamidase, α-L-glutamate arylamidase, glycine arylamidase, leucyl-glycine arylamidase and α-D-galactosidase activities are lacking [4]. The type strain is not pathogenic for hamsters [1].

### Chemotaxonomy

Information on peptidoglycan composition, major cell wall sugars, fatty acids, menaquinones and polar lipids is not available. The mol% G+C of DNA was originally reported to be approximately 48% [3], significantly less than the G+C content inferred from the genome sequence.

## Genome sequencing and annotation

### Genome project history

This organism was selected for sequencing on the basis of its phylogenetic position [40], and is part of the *** G****enomic* ***E****ncyclopedia of* ***Bacteria**** and* ***Archaea***** project [41]. The genome project is deposited in the Genomes On Line Database [21] and the complete genome sequence is deposited in GenBank. Sequencing, finishing and annotation were performed by the DOE Joint Genome Institute (JGI) using state of the art sequencing technology [42]. A summary of the project information is shown in Table 2.

**Table 2:** Genome sequencing project information

**MIGS ID**	**Property**	**Term**
MIGS-31	Finishing quality	Finished
MIGS-28	Libraries used	Five genomic libraries: 454 standard library, 454 PE libraries (3 kb, 4kb and 11 kb insert size), one Illumina library
MIGS-29	Sequencing platforms	Illumina GAii, 454 GS FLX Titanium
MIGS-31.2	Sequencing coverage	1,675.1 × Illumina; 47.0 × pyrosequence
MIGS-30	Assemblers	Newbler version 2.3-PreRelease-6/30/2009, Velvet 1.0.13, phrap version SPS - 4.24
MIGS-32	Gene calling method	Prodigal 1.4, GenePRIMP
	INSDC ID	CP002959 (chromosome) CP002960 (plasmid)
	GenBank Date of Release	June 12, 2012
	GOLD ID	Gc02242
	NCBI project ID	50821
	Database: IMG	2506520013
MIGS-13	Source material identifier	DSM 21527
	Project relevance	Tree of Life, GEBA

### Growth conditions and DNA isolation

*T. parva* strain H^T^, DSM 21527, was grown in semisolid DSMZ medium 1113 (*Leptospira* medium) [43] at 30°C. DNA was isolated from 1-1.5 g of cell paste using MasterPure Gram-positive DNA purification kit (Epicentre MGP04100) following the standard protocol as recommended by the manufacturer with modification st/DL for cell lysis as described in Wu *et al*. 2009 [41]. DNA is available through the DNA Bank Network [44].

### Genome sequencing and assembly

The genome was sequenced using a combination of Illumina and 454 sequencing platforms. All general aspects of library construction and sequencing can be found at the JGI website [45]. Pyrosequencing reads were assembled using the Newbler assembler (Roche). The initial Newbler assembly consisting of 217 contigs in 1 scaffold was converted into a phrap [46] assembly by making fake reads from the consensus, to collect the read pairs in the 454 paired end library. Illumina GAii sequencing data (8,018.4 Mb) was assembled with Velvet [47] and the consensus sequences were shredded into 1.5 kb overlapped fake reads (shreds) and assembled together with the 454 data. The 454 draft assembly was based on 200.6 Mb 454 draft data and all of the 454 paired end data. Newbler parameters are -consed -a 50 -l 350 -g -m -ml 21. The Phred/Phrap/Consed software package [46] was used for sequence assembly and quality assessment in the subsequent finishing process. After the shotgun stage, reads were assembled with parallel phrap (High Performance Software, LLC). Possible mis-assemblies were corrected with gapResolution [45], Dupfinisher [48], or sequencing cloned bridging PCR fragments with subcloning. Gaps between contigs were closed by editing in Consed, by PCR and by Bubble PCR primer walks (J.-F. Chang, unpublished). A total of 361 additional reactions and 11 shatter library were necessary to close some gaps and to raise the quality of the final contigs. Illumina reads were also used to correct potential base errors and increase consensus quality using a software Polisher developed at JGI [49]. The error rate of the final genome sequence is less than 1 in 100,000. Together, the combination of the Illumina and 454 sequencing platforms provided 1,722.1 × coverage of the genome. The final assembly contained 348,698 pyrosequence and 97,925,368 Illumina reads.

### Genome annotation

Genes were identified using Prodigal [50] as part of the DOE-JGI annotation pipeline [51], followed by a round of manual curation using the JGI GenePRIMP pipeline [52]. The predicted CDSs were translated and used to search the National Center for Biotechnology Information (NCBI) nonredundant database, UniProt, TIGR-Fam, Pfam, PRIAM, KEGG, COG, and InterPro databases. Additional gene prediction analysis and functional annotation was performed within the Integrated Microbial Genomes - Expert Review (IMG-ER) platform [53].

## Genome properties

**Figure 3:**
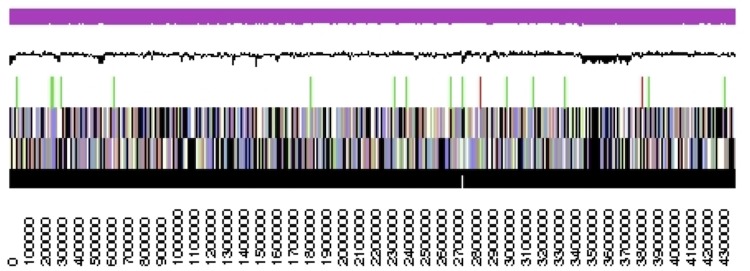
Graphical map of the largest scaffold (smaller scaffold not shown). From bottom to the top: Genes on forward strand (color by COG categories), Genes on reverse strand (color by COG categories), RNA genes (tRNAs green, rRNAs red, other RNAs black), GC content, GC skew (purple/olive).

The genome statistics are provided in Table 3 and Figure 3. The genome in its current assembly consists of two linear scaffolds with a total length of 4,384,015 bp and 25,287 bp, respectively, and a G+C content of 53.6%. Of the 4,214 genes predicted, 4,169 were protein-coding genes, and 45 RNAs; 30 pseudogenes were also identified. The majority of the protein-coding genes (57.9%) were assigned a putative function while the remaining ones were annotated as hypothetical proteins. The distribution of genes into COGs functional categories is presented in Table 4.

**Table 3:** Genome Statistics

**Attribute**	**Value**	**% of Total**
Genome size (bp)	4,409,302	100.00
DNA coding region (bp)	4,062,544	92.14
DNA G+C content (bp)	2,364,784	53.63
Number of scaffolds	2	
Extrachromosomal elements	0	
Total genes	4,214	100.00
RNA genes	45	1.07
rRNA operons	2	
tRNA genes	38	0.90
Protein-coding genes	4,169	98.93
Pseudo genes	30	0.71
Genes with function prediction	2,446	58.04
Genes in paralog clusters	1,807	42.88
Genes assigned to COGs	2,698	64.02
Genes assigned Pfam domains	2,897	68.75
Genes with signal peptides	508	12.06
Genes with transmembrane helices	1,034	24.54
CRISPR repeats	0	

**Table 4:** Number of genes associated with the general COG functional categories

**Code**	**Value**	**% age**	**Description**
J	164	5.5	Translation, ribosomal structure and biogenesis
A	0	0.0	RNA processing and modification
K	169	5.7	Transcription
L	158	5.3	Replication, recombination and repair
B	2	0.1	Chromatin structure and dynamics
D	34	1.2	Cell cycle control, cell division, chromosome partitioning
Y	0	0.0	Nuclear structure
V	49	1.7	Defense mechanisms
T	266	9.0	Signal transduction mechanisms
M	222	7.5	Cell wall/membrane/envelope biogenesis
N	80	2.7	Cell motility
Z	0	0.0	Cytoskeleton
W	0	0.0	Extracellular structures
U	70	2.4	Intracellular trafficking, secretion, and vesicular transport
O	114	3.9	Posttranslational modification, protein turnover, chaperones
C	158	5.3	Energy production and conversion
G	123	4.2	Carbohydrate transport and metabolism
E	154	5.2	Amino acid transport and metabolism
F	73	2.5	Nucleotide transport and metabolism
H	117	4.0	Coenzyme transport and metabolism
I	146	4.9	Lipid transport and metabolism
P	121	4.1	Inorganic ion transport and metabolism
Q	55	1.9	Secondary metabolites biosynthesis, transport and catabolism
R	405	13.7	General function prediction only
S	279	9.4	Function unknown
-	1,516	36.0	Not in COGs

### Emended description of the species *Turneriella parva* Levett *et al.* 2005

The description of the species *Turneriella parva* is the one given by Levett *et al.* 2005 [1], with the following modification: DNA G+C content is 53.6 mol%.
